# Conservation status of polar bears (*Ursus maritimus*) in relation to projected sea-ice declines

**DOI:** 10.1098/rsbl.2016.0556

**Published:** 2016-12

**Authors:** Eric V. Regehr, Kristin L. Laidre, H. Resit Akçakaya, Steven C. Amstrup, Todd C. Atwood, Nicholas J. Lunn, Martyn Obbard, Harry Stern, Gregory W. Thiemann, Øystein Wiig

**Affiliations:** 1 Marine Mammals Management, US Fish and Wildlife Service, Anchorage, AK 99503, USA; 2 Polar Science Center, Applied Physics Laboratory, University of Washington, Seattle, WA 98105, USA; 3 Department of Ecology and Evolution, Stony Brook University, Stony Brook, NY 11794, USA; 4 Polar Bears International, Bozeman, MT 59772, USA; 5 Alaska Science Center, US Geological Survey, Anchorage, AK 99508, USA; 6 Environment and Climate Change Canada, Edmonton, Alberta, Canada T6G 2E9; 7 Ontario Ministry of Natural Resources and Forestry, Peterborough, Ontario, Canada K9J 7B8; 8 Faculty of Environmental Studies, York University, Toronto, Ontario, Canada M3J 1P3; 9 Natural History Museum, University of Oslo, Oslo 0318, Norway

**Keywords:** Arctic, climate change, polar bear, population projections, red list, sea ice

## Abstract

Loss of Arctic sea ice owing to climate change is the primary threat to polar bears throughout their range. We evaluated the potential response of polar bears to sea-ice declines by (i) calculating generation length (GL) for the species, which determines the timeframe for conservation assessments; (ii) developing a standardized sea-ice metric representing important habitat; and (iii) using statistical models and computer simulation to project changes in the global population under three approaches relating polar bear abundance to sea ice. Mean GL was 11.5 years. Ice-covered days declined in all subpopulation areas during 1979–2014 (median −1.26 days year^−1^). The estimated probabilities that reductions in the mean global population size of polar bears will be greater than 30%, 50% and 80% over three generations (35–41 years) were 0.71 (range 0.20–0.95), 0.07 (range 0–0.35) and less than 0.01 (range 0–0.02), respectively. According to IUCN Red List reduction thresholds, which provide a common measure of extinction risk across taxa, these results are consistent with listing the species as vulnerable. Our findings support the potential for large declines in polar bear numbers owing to sea-ice loss, and highlight near-term uncertainty in statistical projections as well as the sensitivity of projections to different plausible assumptions.

## 1. Introduction

**Figure 1.**
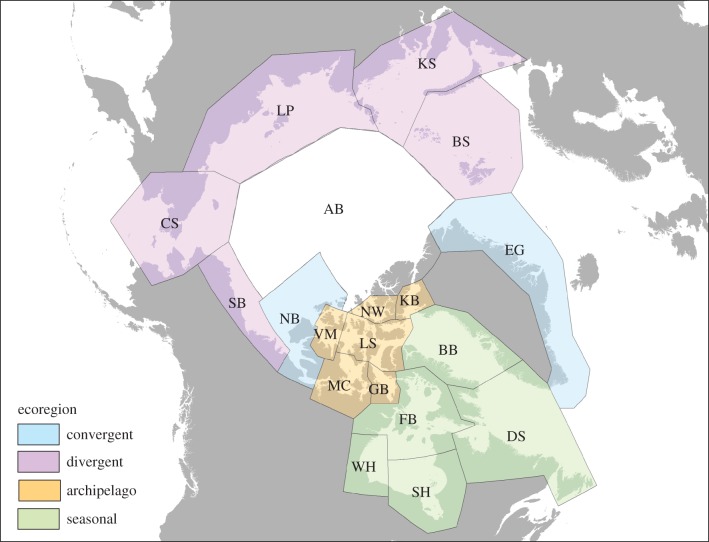
The four polar bear ecoregions and 19 subpopulations. Convergent ecoregion: East Greenland (EG) and Northern Beaufort Sea (NB). Divergent ecoregion: Southern Beaufort Sea (SB), Chukchi Sea (CS), Laptev Sea (LP), Kara Sea (KS) and Barents Sea (BS). Archipelago ecoregion: M'Clintock Channel (MC), Viscount Melville Sound (VM), Norwegian Bay (NW), Kane Basin (KB), Lancaster Sound (LS) and Gulf of Boothia (GB). Seasonal ecoregion: Western Hudson Bay (WH), Foxe Basin (FB), Baffin Bay (BB), Davis Strait (DS) and Southern Hudson Bay (SH). The Arctic Basin (AB) subpopulation likely has few year-round resident polar bears and was excluded from analyses (see electronic supplementary material).

Polar bears (*Ursus maritimus*) depend on sea ice for most aspects of their life history [1]. Anthropogenic climate change is the primary threat to the species because, over the long term, global temperatures will increase and Arctic sea ice will decrease as long as atmospheric greenhouse gas concentrations continue to rise [2,3]. The global population of approximately 26 000 polar bears [4] is divided into 19 subpopulations, which are grouped into four ecoregions reflecting sea-ice dynamics and polar bear life history (figure 1; [5]). The subpopulations currently exhibit variable status relative to climate change [6]. Two have already experienced sea-ice-related demographic declines [7,8]. Others show signs of nutritional stress [9], have been reported as stable or productive [10] or have unknown status owing to deficient data [11].

Methods to forecast the effects of continued sea-ice loss on polar bears have included structured elicitation of expert opinion [12], Bayesian network models evaluating cumulative stressors [5], demographic projections for individual subpopulations [8] and mechanistic models linking vital rates to environmental factors [13]. To date, there has been no global assessment of polar bear abundance data relative to sea ice. We explored future changes in mean global population size (MGPS) for the species, using population projections under three approaches. Approach 1 reflected the hypothesis that environmental carrying capacity (*K*) is directly proportional to the availability of sea ice. Approaches 2 and 3 estimated relationships between changes in sea ice and observed changes in polar bear abundance. We evaluated projection outcomes, over three polar bear generations, relative to thresholds for threatened categories under criterion A3 of the IUCN Red List of Threatened Species (hereafter Red List; [4,14]). The scientific basis of Red List categories is discussed by Mace *et al*. [15].

## 2. Methods

Projection timeframes can incorporate biological differences across species by referencing to generation length (GL, the average age of parents of the current cohort; [14]). We estimated GL as the mean age of adult female polar bears with new cubs based on live-capture data from 11 subpopulations. Females with 1 year-old cubs in year *t* + 1 were counted as pseudo-observations in year *t*. Variation in GL was evaluated, using a bootstrap procedure (electronic supplementary material, table S1).

**Figure 2.**
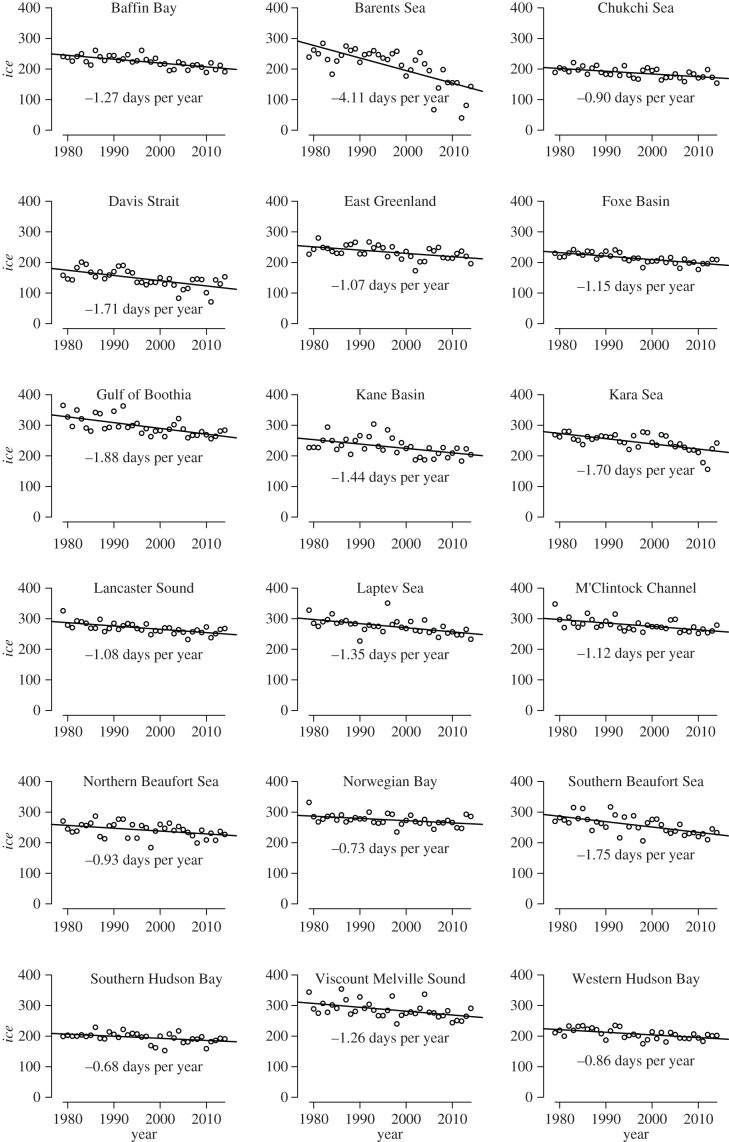
Trends in the standardized sea-ice metric (*ice*), representing important habitat for polar bears, within each subpopulation area during 1979–2014.

Satellite data of sea-ice concentration were collected between years 1979–2014 to develop an index of *K* for polar bears (figure 2; [16]). Within each of the 19 subpopulation areas, daily sea-ice area was calculated by summing the product of ice concentration and grid cell area over all 25 × 25 km grid cells with concentration more than 15%. We then determined the midpoint between summer-minimum and winter-maximum ice areas, and calculated the metric *ice* as the number of days per year that ice area was above the midpoint (i.e. the number of ‘ice-covered’ days). Mean values of *ice* were projected forward, using linear models, which facilitated projections at the spatial scale of polar bear subpopulations (electronic supplementary material, table S2).

We used population projections to evaluate changes in MGPS between the years 2015 and (2015 + 3 × GL) based on three approaches relating *ice* to estimates of subpopulation abundance (*N*; electronic supplementary material, table S3). Approach 1 assumed a one-to-one proportional relationship between *ice* and *N*. Approaches 2 and 3 estimated linear relationships between *ice* and proportional changes in *N*, and used these relationships to predict future values of *N* as a function of projected *ice*. Approach 2 estimated a global *ice*–*N* relationship based on a maximum of two estimates of *N* per subpopulation, separated by at least 10 years, which were available for seven subpopulations. Approach 3 estimated a separate *ice*–*N* relationship for each polar bear ecoregion using a dataset that was similar to approach 2 but included longer time series of *N* available for four subpopulations. All approaches assumed that changes in *N* were mediated primarily through changes in *K* or density-independent habitat effects, and that the ratio *N*/*K* was stable relative to other factors [17]. These assumptions were established on the basis that polar bears depend fundamentally on sea ice, that sea-ice changes represent the main source of habitat modification for the species [5], and that other potential stressors are either secondary (e.g. contaminants; [5]) or have been managed (e.g. harvest; [6]) for most subpopulations in recent decades. Projected subpopulation-specific changes in *N* were scaled to changes in MGPS, using the most recent estimate of *N* for each subpopulation. Based on 62 500 stochastic projections, we calculated the most likely change in MGPS over three generations. In addition, the probabilities of exceeding 0%, 30%, 50% and 80% reduction thresholds were generated following Red List guidelines [14]. We performed computations in R [18], using the package ‘arm’ [19] to simulate uncertainty in model coefficients. Data and projection methods are described fully in the electronic supplementary material.

## 3. Results and discussion

The mean subpopulation-specific estimate of GL was 11.5 years (approx. 5th and 95th percentiles = 9.8 and 13.6, respectively) based on 3374 observed reproductive events (electronic supplementary material, table S1). Projections were performed using GL = 11.5 and 13.6 years to reflect variation in GL and approximate natural GL. We did not apply the lower fifth percentile, because harvest likely shortened several empirical estimates of GL [14]. The metric *ice* declined at a significance level of 0.05 in all 19 subpopulation areas during 1979–2014 (figure 2, median −1.26 days year^−1^ [95% CI = −3.37 to −0.71]; electronic supplementary material, table S2).

We simulated per cent change in MGPS for six scenarios representing two values of GL and three approaches relating *ice* and *N* (table 1). Using GL = 11.5 years, the most likely values for per cent change in MGPS over three generations were −30%, −4% and −43% for approaches 1, 2 and 3, respectively. Across scenarios, the estimated median probabilities of reductions greater than 30%, 50% and 80% in MGPS were 0.71 (range 0.20–0.95), 0.07 (range 0–0.35) and less than 0.01 (range 0–0.02), respectively.

**Table 1.** Simulation results for per cent change in the mean global population size of polar bears.

approach for projections^a^	duration of three polar bear generations (years)	per cent change in mean global population size			probability of decline			
		median	lower 95%CI	upper 95%CI	≥0%	≥30%	≥50%	≥80%
1	35	−30	−35	−25	1.00	0.56	0.00	0.00
1	41	−34	−40	−29	1.00	0.95	0.00	0.00
2	35	−4	−62	50	0.55	0.20	0.06	0.00
2	41	−4	−68	56	0.55	0.24	0.08	0.00
3	35	−43	−76	−20	1.00	0.86	0.30	0.01
3	41	−45	−79	−21	1.00	0.88	0.35	0.02

^a^Approach 1 assumed a one-to-one proportional relationship between sea ice and abundance. Approaches 2 and 3 estimated global and ecoregion-specific relationships between sea ice and empirical estimates of abundance, respectively. Results from each approach are shown for the mean and 95th percentile of estimated GL.

Our analyses highlight the potential for large reductions in MGPS as climate change and sea-ice loss continue [20] over the next three polar bear generations. Approach 1 was based only on projected changes in habitat, a common method when population data are lacking [14]. Approach 2 estimated a global *ice*–*N* relationship that was near 0 and not statistically significant (estimated slope coefficient [*β*] < 0.001, s.e. = 0.005; electronic supplementary material, table S4). This finding reflects variability in current subpopulation status, uncertainty in estimates of *N* and the lack of empirical evidence for sea-ice mediated changes in global abundance over recent decades [6]. Approach 3 estimated a separate *ice*–*N* relationship for each ecoregion. Relationships were positive at a significance level of 0.01 for the seasonal (*β* = 0.013, s.e. = 0.002) and divergent ecoregions (*β* = 0.032, s.e. = 0.009), reflecting observed correlations between declining sea ice and declining abundance (electronic supplementary material, table S4). Relationships were not significant for the convergent (*β* = −0.008, s.e. = 0.009) and archipelago ecoregions (*β* = −0.029, s.e. = 0.030). Although approach 3 reflected regional variability in sea-ice dynamics and polar bear ecology, it was strongly influenced by several well-studied subpopulations and did not reflect finer-scale variation. For example, within the divergent ecoregion, multiple estimates of *N* were available for the declining Southern Beaufort sea subpopulation [7], but not for the Chukchi sea subpopulation, which inhabits a more biologically productive region and has exhibited high recruitment despite sea-ice loss [9].

Our projections (table 1) are broadly consistent with expert opinion [12] and Bayesian network model forecasts [5], although methodological differences preclude direct comparison (see electronic supplementary material). Following the Red List guidelines for risk tolerance ([14]; §3.2.3), the high probability of reductions more than 30% in MGPS, and low probability of reductions more than 50%, were consistent with a categorization of vulnerable (i.e. facing a high risk of extinction in the wild; [4]). Our use of statistical models required estimating few parameters, consistent with sparse data available for Arctic marine mammals [21], and propagated the effects of assumptions on model outcomes in a transparent manner. Future global population assessments could explore the use of hierarchical models [22], integrate data from multiple sources [23], model population processes (e.g. density-dependent interactions between harvest and habitat loss; [17]), consider cumulative effects on polar bear health [24] or consider nonlinear or spatial responses [25].

## Supplementary Material

Electronic Supplementary Material for Regehr et al. 2016

## Data accessibility

The datasets supporting this article have been uploaded as part of the electronic supplementary material.
