# Supplementary material for: Conservation status of polar bears (Ursus maritimus) in relation to projected sea-ice declines
Source: Biol Lett. 2016 Dec;12(12):20160556. doi: 10.1098/rsbl.2016.0556 (PMC5206583; doi:10.1098/rsbl.2016.0556)
Supplement: Electronic Supplementary Material for Regehr et al. 2016 [file rsbl20160556supp1.docx]

**Generation length**

We estimated generation length (GL) for polar bears using live-capture data from 11 subpopulations with available data 1967-2013 (Table S1). Polar bears were captured on the sea ice in spring or the land in summer and fall [1]. The reproductive status of an adult female (AF) was determined based on the presence of dependent young categorized as cub-of-the-year (C0), yearlings (C1), or two year-olds (C2). Polar bear age was determined by counting cementum annuli on a vestigial premolar tooth extracted from bears >1 year old [2] or from body size and dentition for polar bears ≤1 year old. We based GL on females only because polar bears are polygynous and genetic studies to determine paternity are rare, whereas maternity was directly observable from live captures. For each subpopulation, GL was calculated as the arithmetic mean of integer age for AFs with one or more C0, based on direct observations in year *t* and pseudo-observations in year *t* + 1, across all years for which data were available. Approximate 95% confidence intervals were determined from 1,000 replicate datasets derived by resampling with replacement [3]. Empirical estimates of GL, derived from 3,374 observed reproductive events (Table S1), were used to reference the timeframe of population projections as described in the main text.

Multiple observations of an individual AF in year *t* were removed, as were instances of a direct observation in year *t*, and a pseudo-observation in year *t* + 1, which corresponded to the same reproductive event. Multiple observations of an individual AF over different years, representing different reproductive events, were retained because excluding previously-captured AFs from the analysis would bias estimates of GL towards a pool of younger, previously un-captured mothers. If the survival of dependent young was correlated with the mother’s age, then inferring maternity based on observations of AFs with C1s could introduce bias into estimates of GL. We reduced potential bias based on relationships between maternal age and the survival, or timing of weaning, of dependent young, by not inferring maternity based on observations of AFs with C2s. We assumed aging errors from analysis of tooth cementum annuli were random. Thus, field estimates of GL should not be biased by these errors, although standard errors could be underestimated. Possible biases include nonrandom aging errors as a function of bear age or subpopulation ecology [4]. Sampling of AFs that were sighted during fieldwork was random with respect to age, and we had no reason to suspect that sightability of AFs was dependent on age. Estimates of GL from field data may be shorter than natural (i.e., undisturbed) generation length due to human-caused removals. The IUCN Red List guidelines [5] recommend use of natural generation length for conservation assessments. We used the approximate mean and 95th percentile of estimated GL for population projections, to reflect the potential for longer natural generation length (i.e., in the absence of human-caused removals) compared to empirical estimates from field data. The 95th percentile of subpopulation-specific estimates of GL was approximately two years longer than the mean. Based on expert opinion, two years would likely account for the effects of human-caused removals on GL, given that removal rates for most subpopulations were believed to be sustainable over the time period during which field data were collected [6]. Matrix-based population models [7] that include age structure can be used to evaluate GL as a function of survival, recruitment, harvest and other extrinsic factors (E.V. Regehr, *unpublished data*).

**Table S1.** Estimated generation length for 11 polar bear subpopulations with available data. Effective sample size includes direct observations and pseudo-observations, with a maximum of one observation per individual adult female per year. In some cases data were not available for all years within the study period.

| **Subpopulation** | **Study Period** | **Effective sample size** | **Mean generation length (years)** | **95% CI lower** | **95% CI upper** |
| --- | --- | --- | --- | --- | --- |
| Baffin Bay | 1992: 1997 and 2009: 2013 | 170 | 11.6 | 11.0 | 12.4 |
| Barents Sea | 1992: 2013 | 298 | 11.8 | 11.3 | 12.4 |
| Chukchi Sea | 1990: 1994 and 2008: 2013 | 106 | 11.3 | 10.5 | 12.3 |
| Davis Strait | 2005: 2007 | 243 | 10.3 | 9.7 | 10.9 |
| East Greenland | 2007: 2008 | 5 | 9.6 | 6.8 | 12.4 |
| Gulf of Boothia | 1995: 2000 | 95 | 12.6 | 11.6 | 13.5 |
| Lancaster Sound | 1993: 1997 | 230 | 13.1 | 12.5 | 13.7 |
| Northern Beaufort Sea | 1972: 2006 | 172 | 11.4 | 10.7 | 12.2 |
| Southern Beaufort Sea | 1967: 2013 | 440 | 10.7 | 10.3 | 11.2 |
| Southern Hudson Bay | 1984: 2009 | 274 | 10.5 | 10.0 | 11.0 |
| Western Hudson Bay | 1968: 2013 | 1,341 | 13.7 | 13.4 | 14.0 |

**Sea ice**

We developed a standardized sea-ice metric to represent the annual availability of important habitat for polar bears in each of the 19 subpopulations (Table S2). The scientific basis for this metric is described fully in Stern & Laidre [8].

In summary, we used the *Sea Ice Concentrations from Nimbus-7 SMMR and DMSP SSM/I-SSMIS Passive Microwave Data* (ref. 9; updated yearly) available from the National Snow and Ice Data Center (NSIDC) in Boulder, CO, USA. The sea-ice concentrations are calculated using the NASA Team algorithm, and are provided on a polar stereographic grid (true at 70°N) with a nominal grid cell size of 25 × 25 km (the cell size varies slightly with latitude). Temporal coverage of sea-ice concentration data is every other day from 26 October 1978 through 9 July 1987, and then daily through 31 December 2014. We used linear interpolation to fill the alternating-day gaps prior to 9 July 1987, and to span a data gap from 3 December 1987 to 13 January 1988.

For each subpopulation, we calculated the mean September sea-ice area (denoted Area_Sept) and the mean March sea-ice area (denoted Area_March) from 1979-2014. Grid cells with daily sea-ice concentration <15% were considered non-ice covered and not counted toward area. The threshold area (denoted T) was chosen as the midpoint of September and March values:

T = Area_Sept + (50%) × (Area_March – Area_Sept)

The rationale is that sea-ice area reaches its annual minimum in September and its annual maximum in March, so the threshold should be a consistent point between the two. We calculated the metric *ice* as the number of days per year (1979-2014) that sea-ice area exceeded the threshold T. We defined *ice* relative to the entire calendar year (i.e., a maximum of 365 days) to avoid assumptions about periods when sea ice is most important to polar bears. We defined *ice* using subpopulation-specific values of T, rather than a fixed-area, to produce a metric that was likely correlated with the availability of suitable habitat for a significant fraction of the polar bears within each subpopulation. We note that trends in *ice* were not sensitive to the choice of a sea-ice concentration threshold when summing sea-ice area over grid cells. Parkinson [10] used the same sea-ice concentration products to calculate the number of ice-covered days per year, and found that trends over the period 1979-2013, and over shorter periods, were similar using 15% and 50% concentration thresholds to separate ice-covered from non-ice-covered grid cells.

The metric *ice* represents an annual measure of habitat quality and area of occupancy for polar bears, and was used in our population projections as an index for environmental carrying capacity (*K*; ref. 7). We projected *ice* forward using linear models fitted to observed values of the metric from 1979-2014, rather than recalculating the metric based on forecasts of sea-ice extent from global climate models. Summer sea-ice loss based on linear projections is generally more rapid than projections based on the ensemble mean of Coupled Model Intercomparison Project Phase 5 (CMIP5) models through mid-century [11, 12], although we did not evaluate how global climate model output would compare to observed values of the metric *ice*. A key advantage of linear projections is that they can be derived for relatively small regions of the Arctic, such as the channels and fjords of the Canadian Arctic Archipelago where several subpopulations occur.

**Table S2.** Estimated slope, standard error (SE), and significance of least-squared regressions fit to the metric *ice* for the 19 polar bear subpopulations, 1979-2014. Significance of the estimated slopes was determined by F-test: *95% and **99%. The polar bear subpopulations and ecoregions are shown in Figure 1. Annual values of *ice* and the linear trends are shown in Figure 2.

| **Subpopulation** | **Ecoregion** | **Slope (days/year)** | **SE** | **Significance** |
| --- | --- | --- | --- | --- |
| Arctic Basin | Convergent | -2.46 | 0.277 | ** |
| Baffin Bay | Seasonal | -1.27 | 0.216 | ** |
| Barents Sea | Divergent | -4.11 | 0.664 | ** |
| Chukchi Sea | Divergent | -0.90 | 0.213 | ** |
| Davis Strait | Seasonal | -1.71 | 0.367 | ** |
| East Greenland | Convergent | -1.07 | 0.308 | ** |
| Foxe Basin | Seasonal | -1.15 | 0.190 | ** |
| Gulf of Boothia | Archipelago | -1.88 | 0.368 | ** |
| Kane Basin | Archipelago | -1.44 | 0.416 | ** |
| Kara Sea | Divergent | -1.70 | 0.335 | ** |
| Lancaster Sound | Archipelago | -1.08 | 0.216 | ** |
| Laptev Sea | Divergent | -1.35 | 0.338 | ** |
| M’Clintock Channel | Archipelago | -1.12 | 0.274 | ** |
| Northern Beaufort Sea | Convergent | -0.93 | 0.328 | * |
| Norwegian Bay | Archipelago | -0.73 | 0.263 | ** |
| Southern Beaufort Sea | Divergent | -1.75 | 0.363 | ** |
| Southern Hudson Bay | Seasonal | -0.68 | 0.239 | ** |
| Viscount Melville Sound | Archipelago | -1.26 | 0.391 | ** |
| Western Hudson Bay | Seasonal | -0.86 | 0.217 | ** |

**Population projections**

Empirical estimates of subpopulation abundance (*N*) were compiled from published and unpublished sources (Table S3). There is no estimate of *N* for the Arctic Basin subpopulation, which was excluded from population projections. This was unlikely to have a significant effect on projection results because polar bear densities in the Arctic Basin are thought to be low and reflect seasonal or occasional use by bears with fidelity to adjacent subpopulations [6]. The mean coefficient of variation (CV) for estimates of *N* in Table S3 was 0.21. Estimates that did not include a quantitative assessment of uncertainty were assigned a CV of 0.50 for calculations. Estimates that were available as a range were assigned to the midpoint of the range. If a mean estimate of *N* was available over a multiyear period, it was assumed to be valid in the last year of the period.

For population projections under Approach 2, we used a maximum of two estimates of *N* per subpopulation. If multiple estimates of *N* were available, we selected two that were most comparable (e.g., based on a similar geographic area) and separated by at least a decade, and thus represented the mean numerical response of polar bears over a significant portion of the period 1979-2014 during which sea-ice data were available. Using these criteria, 11 subpopulations had a single estimate of *N* and seven subpopulations had two estimates (Table S3). Approach 3 retained the two estimates of *N* (similar to Approach 2) for subpopulations without additional data available, and incorporated longer time series of estimates of *N* for the following relatively well-studied subpopulations: Southern Beaufort Sea (estimates of *N* available 2002-2010; ref. 13), Southern Hudson Bay (1985-1986, 2003-2005; ref. 14), Northern Beaufort Sea (1979, 1985-1989, 2000, 2003-2006; ref. 15), and Western Hudson Bay (1987-2011; ref. 16).

We used three analytical approaches to project polar bear subpopulations and evaluate percent change in mean global population size (MGPS). Approach 1 estimated the proportional change in *N* for subpopulation *i* based solely on predicted values of mean *ice* for subpopulation *i*. For each subpopulation, we took the linear regression model for *ice* and simulated confidence intervals for the model coefficients using the methods of Gelman & Hill [17]. Simulated confidence intervals did not include uncertainty in residual standard errors, and therefore represented uncertainty in predicted mean *ice* rather than the higher level of uncertainty in predicted individual realizations of *ice*. For each draw of the linear model coefficients, we predicted correlated values of mean *ice* for the years 2015 and *j* = (2015 + 3 × GL). We then derived an indicator for the proportional change in abundance of subpopulation *i* as: ∆*N_i,j_* = ( *ice_i,j_* - *ice_i,2015_* ) / | *ice_i,2015_* |.

Approach 2 was similar to Approach 1 except that we included an additional step of estimating a relationship between *ice* and *N*, instead of assuming a one-to-one proportional change. Specifically, we used the dataset in Table S3 to fit a linear model with normalized estimates of *N* (denoted *N^norm^*) as the response variable, and fitted values of *ice* (denoted *fitted.ice*, obtained from the subpopulation-specific regressions of *ice* vs. year) as the predictor variable. Normalization was performed separately for each subpopulation *i* as follows: $N_{i}^{norm}=N_{i}/N_{i}^{1}$, where *N_i_* is either the first or second available abundance estimate, and $N_{i}^{1}$ is the first available abundance estimate. This scaled the first estimate of *N* for each subpopulation to 1 and expressed the second estimate, if available, relative to 1. The effect was that proportional changes in *N* were related to changes in *ice*, regardless of differences in the absolute abundance of the 19 subpopulations (e.g., under this approach, one less ice-covered day resulted in an X% change in subpopulation abundance, rather than a change of Y bears). For a given subpopulation, the two estimates of *N* were assumed to be independent on the basis of being separated by over a decade and, in some cases, resulting from different study methods (Table S3). We used reciprocals of the variances of *N^norm^* as weights in the fitting process to account for differences in sampling uncertainty. Variances of *N^norm^* were estimated from the variances of *N* using the delta method. The linear model for Approach 2 included an intercept for each subpopulation and a single, global slope coefficient (***N^norm^*** = α_BB_ + α_BS_ + α_CS_ … α_WH_ + β_global_ × ***fitted.ice*** + ε; subpopulation abbreviations defined in Table S3). Confidence intervals for model coefficients were simulated using 250 independent draws. For each subpopulation, we randomly selected 250 sets of predicted *ice* values for the years 2015 and *j*, from the 62,500 sets generated under Approach 1. For each set of *ice* values, we predicted correlated values of mean *N* for the years 2015 and *j* using the simulated model coefficients. We then estimated the proportional change in abundance of subpopulation *i* as: ∆*N_i,j_* = (*N_i,j_* - *N_i,2015_* ) / | *N_i,2015_* |. The 250 sets of *ice* values, each of which was used to predict 250 sets of *N* values, resulted in 62,500 point estimates of ∆*N_i,j_* for each subpopulation.

Approach 3 differed from Approach 2 in two ways. First, calculations were applied to a dataset created by merging the data in Table S3 with longer time series of estimates of *N* for relatively well-studied subpopulations (see above). For each subpopulation, this dataset included a maximum of one estimate of *N* per year. We reduced year-to-year sampling variability in the longer time series for the SB and WH subpopulations using a three-point moving average with weights ¼, ½, and ¼. The variances of averaged values were calculated from the standard formula for the variance of a sum, taking into account the covariances (e.g., ref. 18). Covariances were calculated from the lag-1 autocorrelation function of the time series, assuming a simple autoregressive (AR-1) model. Second, the linear model fit under Approach 3 included an intercept for each subpopulation and a slope for each of the four polar bear ecoregions (***N^norm^*** = α_BB_ + α_BS_ + α_CS_ … α_WH_ + β_Seasonal_ × **fitted.ice** + β_Convergent_ × **fitted.ice** + β_Divergent_ × **fitted.ice** + β_Archipelago_ × **fitted.ice** + ε). Approach 3 represents the hypothesis that, within a specific ecoregion, the *ice*-*N* relationship for subpopulations with available data (which included a single subpopulation within each of the Convergent, Divergent, and Archipelago ecoregions) applies to all other subpopulations within that ecoregion. Furthermore, Approach 3 assumed that the more numerous and precise estimates of *N* for well-studied subpopulations represented a valid weight of evidence relative to the sparse data for other subpopulations. We expected linear model coefficients (Table S4) and projection outcomes from Approaches 2 and 3 to be characterized by large uncertainty because of sparse data and large sampling error in estimates of *N* for most subpopulations. We expected uncertainty to be smaller for Approach 1 because it assumed a 1:1 proportional relationship between *ice* and *N*, rather than relying on empirical estimates of *N* to derive this relationship.

Scaling projected subpopulation-specific changes in abundance to percent change in future MGPS requires consideration of the current abundance estimates for each subpopulation. We used the most recent estimate of *N* for each subpopulation (Table S3) as its starting abundance in year 2015 (*N_i,2015_*). Uncertainty in *N_i,2015_* was simulated with 62,500 independent draws from a normal distribution with the estimated mean and standard error for each subpopulation. The lower bound of the distribution was set to 10% of the mean, to preclude implausibly-small starting values. For each draw of starting abundance for subpopulation *i*, we predicted mean abundance in year *j* as: *N_i,j_* = *N_i,2015_* + ∆*N_i,j_* × *N_i,2015_*. We then estimated MGPS in 2015 (MGPS_2015_) and year *j* (MGPS*_j_*) by summing values of *N_i_* over subpopulations. Finally, we calculated percent change in MGPS as: ∆MGPS = 100 × (MGPS*_j_* – MGPS_2015_) / MGPS_2015_. For all approaches, indices of ∆*N_i,j_* were constrained to the interval [-1, 1]. The lower limit of -1 reflects that abundance cannot be negative (i.e., cannot be reduced beyond 100% of its starting value). The upper limit of 1 reflects an assumption that the maximum potential abundance of each subpopulation is approximately two times current abundance. Conceptually, abundance could increase as a subpopulation approaches *K*, or as *K* increases (e.g., for high-latitude subpopulations that were previously limited by heavy ice, it is possible that thinner sea ice would result in transient increases in *K* due to increased biological productivity, improved access of polar bears to seals, or immigration of polar bears from other subpopulations; ref. 19). For all approaches, results were summarized as the median and 95% confidence intervals of estimated ∆MGPS. We estimated the probability of declines greater than 0%, 30%, 50%, and 80% based on the thresholds for threatened categories under criterion A3 of the IUCN Red List (Table 2.1, ref. 5).

Our overall approach is framed as a sensitivity analysis under which potential outcomes were explored for alternative statistical relationships between polar bears and their habitat. These relationships were established on the basis of expert opinion, published studies, and simplifications made to align the analytical approach with the sparse data available for polar bears [20]. Accurate classification of extinction risk is difficult for short time-series of abundance estimates, particularly in the presence of sampling error and autocorrelated process variation [21]. Furthermore, estimating population declines based on just two estimates of *N* or using linear regression on a time series of *N* can be subject to false positives and false negatives [22]. Approach 1 provides an updated numeric reference for the hypothesis that changes in MGPS, over three polar bear generations, are proportional to changes in *K* as represented by our standardized sea-ice metric. Approaches 2 and 3 assumed that sea-ice dynamics have been the primary driver of changes in past estimates of polar bear abundance, as mediated through changes in *K* or the effects of habitat change on intrinsic growth rate. Approaches 2 and 3 also assumed that relationships between *ice* and *N^norm^* were linear, and that relationships estimated from observed data will persist three generations into the future. By estimating a global relationship between *ice* and *N^norm^*, Approach 2 did not reflect potential spatial patterns in the response of polar bears to ecological change (e.g., that lighter ice conditions could provide transient benefits to bears at higher latitudes, while limiting foraging opportunities at southerly latitudes). On the other hand, Approach 3 estimated ecoregion-specific relationships between *ice* and *N^norm^*. Although Approach 3 used the most comprehensive abundance dataset available, there was only a single subpopulation with abundance data within each of the Convergent, Divergent, and Archipelago ecoregions (Table S4). Therefore, Approach 3 was more strongly influenced, relative to the other approaches, by which subpopulations have been studied. Also it did not account for potential variation in subpopulation status within the Convergent, Divergent, and Archipelago ecoregions. Sparse data precluded analysis of variation in population responses across ecoregions or other groupings.

Our findings are broadly consistent with expert-opinion assessments of the directionality and magnitude of future reductions in the global population of polar bears. Half of the participants in the survey by O’Neill et al. [23] suspected at least a 30% decrease in global population size by 2050. Similarly, in the previous IUCN Red List assessment, Schliebe et al. [24] suspected a population reduction of greater than 30% within three generations (45 years in that analysis) due to declines in area of occupancy, extent of occurrence, and habitat quality. For comparison of those studies to our findings, across the six scenarios in Table 1 the median percent change in MGPS was approximately -32% (range - 45% to -4%). Amstrup et al. [25] used a deterministic model to relate *K* for polar bears to forecasts of sea-ice availability from global climate models. Depending on the climate model used, that method suggested a 10%-22% reduction in global *K* after 45 years, with the most pronounced declines in the Divergent and Seasonal ecoregions. Amstrup et al. [25, 26, 27] and Atwood et al. (2016) used successive generations of a Bayesian Network model to evaluate and rank long-term threats to the persistence of polar bears. Atwood et al. [28] suggested that polar bears could reach a dominant probability of a “greatly decreased” state (defined as occurring in reduced numbers or distribution that make polar bears difficult to detect and vulnerable to stressors) in the Divergent ecoregion by 2030, and perhaps as soon as 2055 in the Seasonal and Convergent ecoregions. Projections for the Archipelago ecoregion were less uniform, with a significant probability of remaining in a “same” or “decreased” state by the end of the 21st century. Qualitatively, the projected status of the four ecoregions would be ranked similarly based on the probability of decline in Atwood et al. [28], and based on the sign and magnitude of ecoregion-specific slopes under Approach 3 in our calculations (Table S4). Different analytical frameworks and approaches to interpreting outcomes make direct comparison of our analysis with a Bayesian Network model difficult. Furthermore, Atwood et al. [28] considered a large suite of potential stressors and interactions among stressors, whereas our approach evaluated statistical (i.e., non-mechanistic) relationships between *ice* and *N*.

**Table S3.** Abundance estimates for the 19 polar bear subpopulations. The Method column indicates capture-recapture study (CR), den count (DC), distance sampling (DS), expert opinion (EO), and other (O). NA indicates that estimates were not available.

| **Subpopulation** | **Abbreviation** | **Year** | **Estimate** | **95%_lwr** | **95%_uwr** | **Method** | **Reference** |
| --- | --- | --- | --- | --- | --- | --- | --- |
| Arctic Basin | AB | NA | NA | NA | NA | NA | 6 |
| Baffin Bay | BB | 1997 | 2,074 | 1,553 | 2,595 | CR | 29 |
| Barents Sea | BS | 2004 | 2,644 | 1,899 | 3,592 | DS | 30 |
| Chukchi Sea | CS | 1997 | 2,000 | NA | NA | EO | 31 |
| Davis Strait | DS | 1996 | 1,400 | NA | NA | EO | 32 |
| Davis Strait | DS | 2007 | 2,158 | 1,833 | 2,542 | CR | 33 |
| East Greenland | EG | 1997 | 2,000 | NA | NA | EO | 31 |
| Foxe Basin | FB | 1994 | 2,197 | 1,677 | 2,707 | CR | 34 |
| Foxe Basin | FB | 2010 | 2,580 | 2,093 | 3,180 | DS | 35 |
| Gulf of Boothia | GB | 1986 | 900 | NA | NA | EO | 36 |
| Gulf of Boothia | GB | 2000 | 1,592 | 870 | 2,314 | CR | 37 |
| Kane Basin | KB | 1997 | 164 | 94 | 234 | CR | 38 |
| Kara Sea | KS | 2013 | 3,200 | NA^a^ | NA | O | 39 |
| Lancaster Sound | LS | 1997 | 2,541 | 1,759 | 3,323 | CR | 40 |
| Laptev Sea | LP | 1993 | 1,000 | NA | NA | DC/EO | 41 |
| M’Clintock Channel | MC | 2000 | 284 | 166 | 402 | CR | 42 |
| Northern Beaufort Sea | NB | 1979 | 876 | 1^b^ | 1,844 | CR | 15 |
| Northern Beaufort Sea | NB | 2006 | 1,004 | 1^b^ | 2,062 | CR | 15 |
| Norwegian Bay | NW | 1997 | 203 | 115 | 291 | CR | 40 |
| Southern Beaufort Sea | SB | 1986 | 1,800 | NA | NA | CR | 43 |
| Southern Beaufort Sea | SB | 2010 | 907 | 548 | 1,270 | CR | 13 |
| Southern Hudson Bay | SH | 1986 | 1,000^c^ | 367 | 1,633 | CR/EO | 44 |
| Southern Hudson Bay | SH | 2012 | 943 | 658 | 1,350 | DS | 45 |
| Viscount Melville Sound | VM | 1992 | 161 | 121 | 201 | CR | 46 |
| Western Hudson Bay | WH | 1987 | 1,194 | 1,020 | 1,368 | CR | 47 |
| Western Hudson Bay | WH | 2011 | 1,030 | 754 | 1,406 | DS | 48 |

^a^Estimates of uncertainty for the Kara Sea subpopulation were not used due to questions about the statistical methods in Matishov et al. [39].

^b^Lower bound of the 95% CI set to 1, because it was negative based on the expected value and standard error provided in Stirling et al. [15].

^c^Estimate of approximately 900 bears in Kolenosky et al. [44] was revised to 1,000 by Canadian Polar Bear Technical Committee due to sampling issues [49].

**Table S4.** Estimated slope, standard error (SE), and significance of linear models fit to abundance and sea-ice data. *N^norm^* is normalized subpopulation abundance and *ice* is the standardized sea-ice metric. Significance of slope according to P-test: *95% and **99%. Subpopulation abbreviations are provided in Table S3.

| **Approach** | **Region** | **Slope (N^norm^/*ice*)** | **SE** | **Sig.** | **Number of abundance estimates per subpopulation used to estimate slope coefficient** |
| --- | --- | --- | --- | --- | --- |
| 2 | Global | <0.001 | 0.005 |  | 2 DS, 2 FB, 2 GB, 2 NB, 2 SB, 2 SH, 2 WH |
| 3 | Seasonal | 0.013 | 0.002 | ** | 2 DS, 2 FB, 6 SH, 24 WH |
| 3 | Convergent | -0.008 | 0.009 |  | 10 NB |
| 3 | Divergent | 0.032 | 0.009 | ** | 8 SB |
| 3 | Archipelago | -0.029 | 0.030 |  | 2 GB |

**References**

[1] Stirling, I., Spencer, C. & Andriashek, D. 1989. Immobilization of polar bears (*Ursus maritimus*) with Telazol® in the Canadian Arctic. *Journal of Wildlife Diseases* 25: 159-168.

[2] Calvert, W. & Ramsay, M.A. 1988. Evaluation of age determination of polar bears by counts of cementum growth layer groups. *Ursus* 10: 449-453.

[3] Manly, B.F.J. 1991. *Randomization and Monte Carlo Methods in Biology*. Chapman and Hall , New York, NY, USA.

[4] Christensen-Dalsgaard, S.N., Aars, J., Andersen, M., Lockyer, C. & Yoccoz, N.G. 2010. Accuracy and precision in estimation of age of Norwegian Arctic polar bears (*Ursus maritimus*) using dental cementum layers from known-age individuals. *Polar Biology* 33: 589-597.

[5] IUCN Standards and Petitions Subcommittee. 2016. *Guidelines for Using the IUCN Red List Categories and Criteria. Version 12. Prepared by the Standards and Petitions Subcommittee*. Available from: http://www.iucnredlist.org/documents/RedListGuidelines.pdf. (Accessed 03 October 2016)

[6] Obbard, M.E., Thiemann, G.W., Peacock, E. & DeBruyn, T.D (eds). 2010. *Polar Bears: Proceedings of the 15th Working Meeting of the IUCN/SSC Polar Bear Specialist Group, Copenhagen, Denmark, 29 June–3 July 2009*. pp. 235. IUCN, Gland, Switzerland and Cambridge, UK.

[7] Regehr E.V., Wilson R.R., Rode K.D. & Runge M.C. 2015. Resilience and risk—A demographic model to inform conservation planning for polar bears. *U.S. Geological Survey Open-File Report* 2015-1029, 56. (doi:http://dx.doi.org/10.3133/ofr20151029).

[8] Stern H.S. & Laidre K.L. 2016. Sea-Ice indicators of polar bear habitat. *The Cryosphere* 10: 2027-2041.

[9] Cavalieri, D. J., Parkinson, C. L., Gloersen, P., & Zwally, H. J. 1996. Sea Ice Concentrations from Nimbus-7 SMMR and DMSP SSM/ISSMIS Passive Microwave Data, Version 1, Boulder, Colorado USA, NASA National Snow and Ice Data Center Distributed Active Archive Center, doi:10.5067/8GQ8LZQVL0VL, updated yearly.

[10] Parkinson, C.L. 2014. Spatially mapped reductions in the length of the Arctic sea ice season. *Geophysical Research Letters* 41: 4316-4322.

[11] Stroeve, J.C., Kattsov, V., Barrett, A., Serreze, M., Pavlova, T., Holland, M. & Meier, W.N. 2012. Trends in Arctic sea ice extent from CMIP5, CMIP3 and observations. *Geophysical Research Letters* 39: L16502.

[12] Overland, J.E. & Wang, M. 2013. When will the summer Arctic be nearly sea ice free? *Geophysical Research Letters* 40: 2097-2101.

[13] Bromaghin, J.F., McDonald, T.L., Stirling, I., Derocher, A.E., Richardson, E.S., Regehr, E.V., Douglas, D.C., Durner, G.M., Atwood, T. & Amstrup, S.C. 2015. Polar bear population dynamics in the Beaufort Sea during a period of sea ice decline. *Ecological Applications* 25: 634-651.

[14] Obbard, M.E. McDonald, T.L., Howe, E.J., Regehr, E.V. & Richardson, E.S. 2007. Polar Bear Population Status in Southern Hudson Bay, Canada. U.S. Geological Survey Administrative Report. Reston, VA, USA.

[15] Stirling, I., McDonald, T.L., Richardson, E.S., Regehr, E.V. & Amstrup, S.C. 2011. Polar bear population status in the northern Beaufort Sea, Canada, 1971–2006. *Ecological Applications* 21: 859-876.

[16] Lunn N.J., Servanty S., Regehr E.V., Converse S.J., Richardson E. & Stirling I. 2016. Demography of an apex predator at the edge of its range: impacts of changing sea ice on polar bears in Hudson Bay*. Ecological Applications* 26: 1302-1320.

[17] Gelman, A. & Hill, J. 2006. *Data Analysis Using Regression and Multilevel/Hierarchical Models*. Cambridge University Press, New York, NY, USA.

[18] Arnold, S.F. 1990. *Mathematical Statistics*. Prentice Hall, Englewood Cliffs, NJ, USA.

[19] Derocher, A. E., Lunn, N. J. & Stirling, I. 2004. Polar bears in a warming climate. *Integrated Comparative Biology* 44: 163–176.

[20] Laidre K., Stern H., Kovacs K.M., Lowry L., Moore S.E., Regehr E.V., Ferguson S.H., Wiig Ø., Boveng P., Angliss R.P., et al. 2015. Arctic marine mammal population status, sea ice habitat loss, and conservation recommendations for the 21st century. *Conservation Bio*logy 29: 724-737.

[21] Connors ,B.M., Cooper A.B., Peterman R.M. & Dulvy N.K. 2014. The false classification of extinction risk in noisy environments. *Proceedings of the Royal Society B 281: 20132935* 281: 20132935.

[22] Wilson, H.B., Kendall, B.E. & Possingham, H.P. 2011. Variability in population abundance and the classification of extinction risk. *Conservation Biology* 25: 747-757.

[23] O’Neill, S.J., Osborn, T.J., Hulme, M., Lorenzoni, I. & Watkinson, A.R. 2008. Using expert knowledge to assess uncertainties in future polar bear populations under climate change. *Journal of Applied Ecology* 45: 1649-1659.

[24] Schliebe, S., Wiig, Ø., Derocher, A. & Lunn, N. 2008. Ursus maritimus. The IUCN Red List of Threatened Species. Version 2014.3. Available at: www.iucnredlist.org. (Accessed 06 May 2015).

[25] Amstrup, S. C., Marcot, B.G. & Douglas, D.C. 2007. Forecasting the range-wide status of polar bears at selected times in the 21st century. U.S. Geological Survey Administrative Report. Reston, VA, USA.

[26] Amstrup, S.C., Marcot, B.G. & Douglas, D.C. 2008. A Bayesian network modeling approach to forecasting the 21st century worldwide status of polar bears. In: DeWeaver, E.T., Bitz, C.M. & Tremblay, L.B. (eds), *Arctic Sea Ice Decline: Observations, Projections, Mechanisms and Implications.* , pp. 213-268. Geophysical Monograph Series, American Geophysical Union, Washington, DC, USA.

[27] Amstrup, S.C., DeWeaver, E.T., Douglas, D.C., Marcot, B.G., Durner, G.M., Bitz, C.M. & Bailey, D.A. 2010. Greenhouse gas mitigation can reduce sea-ice loss and increase polar bear persistence. *Nature* 468: 955-958.

[28] Atwood T.C., Marcot B.G., Douglas D.C., Amstrup S.C., Rode K.D., Durner G.M. & Bromaghin JF. 2016. Forecasting the relative influence of environmental and anthropogenic stressors on polar bears. *Ecosphere* 7: e01370.

[29] Taylor, M.K., Laake, J., McLoughlin, P.D., Born, E.W., Cluff, H.D., Ferguson, S.H., Rosing-Asvid, A., Schweinsburg, R. & Messier, F. 2005. Demography and viability of a hunted population of polar bears. *Arctic* 58: 203-214.

[30] Aars, J., Marques, T.A., Buckland, S.T., Andersen, M., Belikov, S., Boltunov, A. & Wiig, Ø. 2009. Estimating the Barents Sea polar bear subpopulation size. *Marine Mammal Science* 25: 35-52.

[31] Lunn, N.J., Schliebe, S. & Born, E.W. (eds), 2002. *Polar Bears: Proceedings of the 14th Working Meeting of the IUCN/SSC Polar Bear Specialist Group, Nuuk, Greenland, 23-28 June 2001*, pp. 21-35. IUCN, Gland, Switzerland and Cambridge, UK.

[32] Derocher, A.E., Garner, G.W., Lunn, N.J. & Wiig, Ø. (eds), 1998. *Polar Bears: Proceedings of the 12th Working Meeting of the IUCN/SSC Polar Bear Specialist Group, Oslo, Norway, 3-7 February 1997*, pp. 23-44. IUCN, Gland, Switzerland and Cambridge, UK.

[33] Peacock, E., Taylor, M.K., Laake, J. & Stirling, I. 2013. Population ecology of polar bears in Davis Strait, Canada and Greenland. *Journal of Wildlife Management* 77: 463-476.

[34] Taylor, M. K., Lee, J., Laake, J. & McLoughlin, P. D. 2006. Estimating population size of polar bears in Foxe Basin, Nunavut using tetracycline biomarkers. Department of the Environment, Government of Nunavut.

[35] Stapleton, S., Peacock, E., Garshelis, D. & Atkinson, S. 2012. Aerial survey population monitoring of polar bears in Foxe Basin. Final project report (2-10-13) to the Nunavut Wildlife Research Trust.

[36] Wiig, Ø., Born, E.W. & Garner, G.W. (eds), 1995. *Polar Bears: Proceedings of the 11th Working Meeting of the IUCN/SSC Polar Bear Specialist Group, Copenhagen, Denmark, 25-27 January 1993*, pp. 19-24. IUCN, Gland, Switzerland and Cambridge, UK.

[37] Taylor, M.K., Laake, J., McLoughlin, P.D., Cluff, H.D. & Messier, F. 2009. Demography and population viability of polar bears in the Gulf of Boothia, Nunavut. . *Marine Mammal Science* 25: 778-796.

[38] Taylor, M.K., Laake, J., McLoughlin, P.D., Cluff, H.D., Born, E.W., Rosing-Asvid, A. & Messier, F. 2008. Population parameters and harvest risks for polar bears (*Ursus maritimus*) of Kane Basin, Canada and Greenland. *Polar Biology* 31: 491-499.

[39] Matishov, G.G., Chelintsev, N.G., Goryaev, Yu.I., Makarevich, P.R. & Ishkulov, D.G. 2014. Assessment of the amount of polar bears (*Ursus maritimus*) on the basis of perennial vessel counts. *Doklady Earth Sciences* 458: 1312-1316.

[40] Taylor, M.K., Laake, J., McLoughlin, P.D., Cluff, H.D. & Messier, F. 2008. Mark-recapture and stochastic population models for polar bears of the High Arctic. *Arctic* 61: 143-152.

[41] Belikov, S.E. & Randla, T.E. 1987. Fauna of birds and mammals of Severnaya Zemlya. In: Syroyechkovskiy, E.E. (ed.), *Fauna and Ecology of Birds and Mammals in Middle Siberia*, pp. 18-28. Nauka, Moscow, USSR.

[42] Taylor, M.K., Laake, J., McLoughlin, P.D., Cluff, H.D. & Messier, F. 2006. Demographic parameters and harvest-explicit population viability analysis for polar bears in M'Clintock Channel, Nunavut, Canada. *Journal of Wildlife Management* 70(1667-1673).

[43] Amstrup, S.C., I. Stirling, & J.W. Lentfer. 1986. Past and present status of polar bears in Alaska. *Wildlife Society Bulletin* 14: 241-254.

[44] Kolenosky, G.B., Abraham, K.F. & Greenwood, C.J. 1992. Polar bears of southern Hudson Bay. Polar Bear Project, 1984-88. *Final Report. Ontario Ministry of Natural Resources*, Maple, ON, Canada.

[45] Obbard, M.E., Middel, K.R., Stapleton, S., Thibault, I., Brodeur, V. & Jutras, C. 2015. Estimating abundance of the Southern Hudson Bay polar bear subpopulation with aerial surveys. *Polar Biology*. DOI 10.1007/s00300-015-1737-5.

[46] Taylor, M.K., Laake, J., Cluff, H.D., Ramsay, M. & Messier, F. 2002. Managing the risk from hunting for the Viscount Melville Sound polar bear population. *Ursus* 13: 185-202.

[47] Regehr, E.V., Lunn, N.J. Amstrup, S.C. & Stirling, I. 2007. Effects of earlier sea ice breakup on survival and population size of polar bears in western Hudson Bay. *Journal of Wildlife Management* 71: 2673-2683.

[48] Stapleton, S., Atkinson, S., Hedman, D. & Garshelis, D. 2014. Revisiting Western Hudson Bay: Using aerial surveys to update polar bear abundance in a sentinel population. *Biological Conservation* 170: 38-47.

[49] PBSG. 2015. Summary of polar bear population status as per 2014: Southern Hudson Bay (SH). Available at: http://pbsg.npolar.no/en/status/populations/southern-hudson-bay.html. (Accessed: 25 January 2015).
